# Cerebral vasomotor reactivity assessment using Transcranial Doppler and MRI with apnea test

**DOI:** 10.1590/1414-431X20165437

**Published:** 2016-10-24

**Authors:** C.R. Herrera Campos, G.C. Beltramini, W.M. Avelar, F.O. Lima, L.M. Li

**Affiliations:** 1 Departamento de Neurologia, Faculdade de Ciências Médicas, Universidade Estadual de Campinas, Campinas, SP, Brasil; 2 Instituto de Física "Gleb Wataghin", Universidade Estadual de Campinas, Campinas, SP, Brasil; 3 Complexo Hospitalar Prefeito Edivaldo Orsi “Ouro Verde”, Campinas, SP, Brasil; 4 Brazilian Research Institute for Neuroscience and Neurotechnology (BRAINN), Universidade Estadual de Campinas, Campinas, SP, Brasil; 5 Universidade de Fortaleza, Fortaleza, CE, Brasil

**Keywords:** Blood oxygen level dependence, Transcranial Doppler, Carotid stenosis

## Abstract

Differently from previous studies that used Transcranial Doppler (TCD) and functional MRI (fMRI) for cerebral vasomotor reactivity (CVR) assessment in patients with carotid stenosis (CS), we assessed CVR using an identical stimulus, the Breath-Holding Test (BHT). We included 15 patients with CS and 7 age-matched controls to verify whether fMRI responded differently to BHT between groups and to calculate the agreement rate between tests. For TCD, impaired CVR was defined when the mean percentage increase on middle cerebral artery velocities was ≤31% on 3 consecutive 30-s apnea intercalated by 4-min normal breathing intervals. For fMRI, the percent variation on blood oxygen level-dependent (BOLD) signal intensity in the lentiform nucleus (LN) ipsilateral to the CS (or both LNs for controls) from baseline breathing to apnea was measured. The Euclidian differences between the series of each subject and the series of controls and patients classified it into normal or impaired CVR. We found different percent variations on BOLD-signal intensities between groups (P=0.032). The agreement was good in Controls (85.7%; κ=0.69) and overall (77.3%; κ=0.54). We conclude that BHT was feasible for CVR assessment on fMRI and elicited different BOLD responses in patients and controls, with a good overall agreement between the tests.

## Introduction

Patients with critical carotid stenosis (CS) may present reduction of the cerebral perfusion pressure when collateral flow is insufficient. This may lead to a vasodilation of resistance arterioles mainly modulated by CO_2_ and, secondly, to a decrease in cerebral blood flow (CBF) and an increase in oxygen extraction fraction (OEF) (1,2). The initial vasodilatory response reflects the cerebral vasomotor reactivity (CVR). Impaired CVR is an independent risk factor for ischemic events in patients with CS (3–5).

Positron emission tomography (PET) is the gold-standard method to assess CVR (1,6). However, due to the wide unavailability and high costs of PET, transcranial Doppler ultrasound (TCD) with transient hypercapnia vasodilatory stimuli such as acetazolamide, inhalation of carbon dioxide or breath holding test (BHT) has been used in clinical practice instead (6–12).

More recently, with the advantage of imaging resolution, blood oxygen level-dependent (BOLD) contrast in functional magnetic resonance imaging (fMRI) has been often used for CVR assessment (13–18). fMRI detects changes in the concentration of deoxyhemoglobin, dependent on a complex interplay among CBF, blood volume and cerebral oxygen consumption (13,19). Hypercapnia increases CBF, which reduces the concentration of deoxyhemoglobin leading to an increase in BOLD signal.

Few studies have associated TCD to BOLD-fMRI for CVR assessment, most of them using distinct vasodilatory stimuli (16 [Bibr B17],^18^,^20^–22). Differently, we aimed to assess CVR in patients with CS and controls using an identical vasodilatory stimulus (BHT) in both tests to verify whether BOLD curves responded differently between the groups and, additionally, to calculate the agreement between the tests.

## Material and Methods

### Subjects

Subjects were recruited from the outpatient Neurology Clinic at the Hospital das Clínicas, Universidade Estadual de Campinas (UNICAMP; patients) or among hospital employees, or from a Community Physical Activity program for the elderly (controls) between October 2010 and January 2013.

Sixteen patients (12 males; mean age: 68 years; range: 52-90) with CS ≥60% by angiography, ≥70% by duplex ultrasound, or ≥80% by computed tomography angiography or MRI angiography (CREST criteria) (23), and 7 age-matched controls with no history of cerebrovascular disease (5 males; mean age: 64.5 years range: 51-85) were enrolled. One patient (76-year-old male; labeled “P12”) was lately excluded from the analysis, as he did not comply with the BHT on fMRI. Therefore, 15 patients were included.

Patients had to be asymptomatic or, if otherwise, have only transient ischemic attack, *Amaurosis fugax* or syncope. Controls were neurologically healthy and had normal carotid arteries, as seen by duplex ultrasound. Subjects with self-declared respiratory disorders were not included. Subjects with LN lesions or diffuse leukoencephalopathy were excluded, as this could affect the BOLD signal assessment in the region of interest (ROI).

Demographics are shown in Table 1. All subjects provided written informed consent. The study was approved by the institutional review board of the Faculdade de Ciências Médicas of UNICAMP.

**Table:**
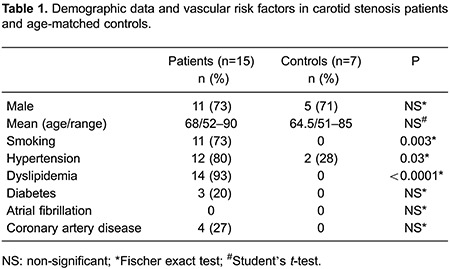

### Transcranial Doppler ultrasound protocol

Subjects were evaluated in a quiet room and in supine position. Throughout the session, arterial blood pressure was measured continuously. Expiratory end-tidal CO_2_ was recorded throughout the experiment through a nasal sampling line attached to a capnometer (Capnocheck™II8401 Smiths Medical, USA).

The M1 segment of both middle cerebral arteries (MCA) were identified through the examination of the transtemporal window with a 2-MHz transducer (SONARA™ Viasys Healthcare, USA) operated by an experienced sonographer (C.R.C.H.). Upon isolating the site of highest flow velocity within the segment, the transducer was held constant through a head frame to measure time-averaged maximum mean velocities (MMV). Lack of a suitable transtemporal window precluded the performance of bilateral examination in 3 controls and in 3 patients with unilateral CS (P01, P08, and P16). In these patients, TCD examination was done ipsilateral to the CS only.

Prior to TCD, all subjects were instructed about BHT, which consisted of an initial 5-min resting time followed by three 30-s periods of transient apnea (verbally coached by the examiner) intercalated by 4-min of normal breathing between them. Subjects were instructed to avoid hyperventilation before breath-holding time as well as to perform a not so deep inspiratory breath-hold in order to prevent a Valsalva episode. After the breath-hold, a quick exhalation of residual air was performed prior to a return to natural breathing, which allowed the measurement of end-tidal CO_2_ increases as a result of the breath-hold. All participants performed BHT in the same way and under continuous examiner supervision.

Time-averaged MMV immediately before (baseline) and after each apnea period were recorded. Impaired CVR was considered if the mean percentage increase on the MCA time-averaged MMV from baseline to apnea was ≤31% or steal phenomenon occurred (8 [Bibr B09] [Bibr B10]).

### Functional MRI protocol

fMRI acquisition was performed with a 3.0T MRI scanner (Philips Achieva, Netherlands) and included: 3D T1-weighted image for anatomical reference (240×240×180 mm^3^ FOV, 1.00×1.00×1.00 mm^3^ voxel size, 240×240×180 matrix, 8° flip angle, TR=6.9 ms, TE=3.2 ms), FLAIR image for visualization of parenchymal lesions (200×185.71×149 mm^3^ FOV, 0.45×0.45×4.00 mm^3^ voxel size, 448×448×30 matrix, 1 mm slice gap, TR=12,000 ms, TE=140 ms, TI=2850 ms), DWI to assess recent subclinical lesions (230×230×119 mm^3^ FOV, 0.90×0.90×4.00 mm^3^ voxel size, 256×256×24 matrix, 1 mm slice gap, 90° flip angle, TR=2198 ms, TE=60 ms, b-value=1000 s/mm^2^) and echo-planar image (EPI) to assess the BOLD effect (240×240×120 mm^3^ FOV, 3.00×3.00×3.00 mm^3^ voxel size, 80×80×40 matrix, no slice gap, 90° flip angle, TR=2000 ms, TE=30 ms).

Prior to scanning, all subjects were instructed to stay as still as possible while on fMRI examination and to look continually at a computer screen (Eloquence, Invivo, USA) positioned on the head coil for breath-hold paradigm instructions. Breathing tasks were displayed in traffic lights-based visual commands: green screen for normal breathing (3 min 50 s); yellow, for normal breathing in preparation for holding (10 s); and red, for an inspiratory breath holding (24 s). This sequence was repeated 3 times in the same EPI acquisition (total: 17 min 12 s), ending with a 4-min green screen. The instructions were the same as done earlier, i.e., to avoid hyperventilation before breath-holding time as well as to perform a not so deep inspiratory breath-hold in order to prevent Valsalva. A respiratory gating belt was used for apnea monitoring.

Functional images were visually examined to detect gross artifacts and preprocessed with SPM8 (http://www.fil.ion.ucl.ac.uk/spm) running on MATLAB R2010a (The MathWorks, Inc., USA). The images were realigned (6-parameter rigid body transformation), normalized to standard Montreal Neurological Institute space using the unified segmentation (24) and spatially smoothed with a Gaussian kernel of 6 mm full width at half maximum.

For the patients, the ROI was the LN ipsilateral to the stenosis. In patients with bilateral CS, the ROI was ipsilateral to the higher-grade stenosis. In controls, both LN were used. LN masks were provided by the WFU PickAtlas toolbox (about 400 voxels each). The extracted signal is the average value of all the voxels inside the ROI. The average signal of both LN was extracted and corrected for motion through a multiple linear regression with 6 motion parameters from preprocessing (25).

**Figure 1:**
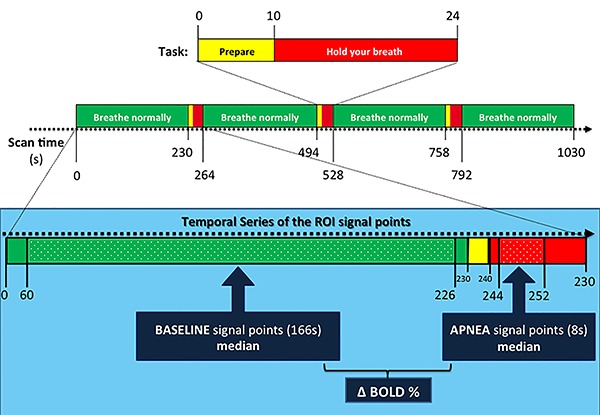
*Top*: visual BOLD-functional MRI activation paradigm based on traffic lights for instructing subjects on breathing tasks during echo-planar image acquisition. *Bottom*, within the light blue rectangle: a detailed view of the temporal series of the region of interest (ROI) signal points that were considered for baseline and apnea signal points median calculation, as well as the for the percent variation in the magnitude of stimulus-induced BOLD signal (delta BOLD%).

To avoid any hemodynamic and respiratory signal changes related either to breath holding or to inspiration or expiration movements, we considered for baseline BOLD signal the instants when the subject was looking at the green screen and excluded 4 s of the signal that preceded the yellow screen presentation and 60 s after the red screen. From this set of baseline signal points, we calculated the median rather than the average in order to avoid the effect of outliers. The stimulus-induced BOLD signal level was calculated through the analysis of the interval from 4 to 12 s after the onset of breath holding, as the series showed greater differences between them at this period. Afterwards, the percent variation in the magnitude of stimulus-induced BOLD signal (delta-BOLD%) was calculated in relation to the baseline median BOLD signal level (Figure 1).

The temporal series of the ROI of controls and patients were individually analyzed by a researcher who was blind to TCD results. Each subject was then classified as normal or abnormal CVR. Since the number of subjects was small, the training data for the classifier was the whole data with the time series of the current subject removed. The average signal across all remaining individuals in each group (controls and patients) was calculated. Then, the Euclidian distance between the series of the current subject and the series of each group was compared. The smallest distance guided the classification: if the distance between the series of a given subject and the series of the controls was smaller than the distance between this subject and the series of patients, then CVR was considered normal.

### Statistical analysis

Patients and controls were classified by each one of the tests into normal or impaired CVR. Afterwards, the agreement was calculated through Cohen's kappa coefficient (κ). Comparisons of nominal data and age distribution between groups were performed with Fisher’s exact test and two-tailed Student’s *t*-test, respectively. The comparison of the delta-BOLD% between groups was done with the Mann-Whitney test. In all tests, P<0.05 was considered to be statistically significant.

## Results

On TCD, impaired CVR was detected in 2 of the 7 controls (men; 70 and 85 years old). The oldest one had well-controlled hypertension; the youngest one had no risk factors. Among patients, 10 of 15 showed impaired CVR. Demographic, TCD and fMRI data are shown in Table 2.

**Table:**
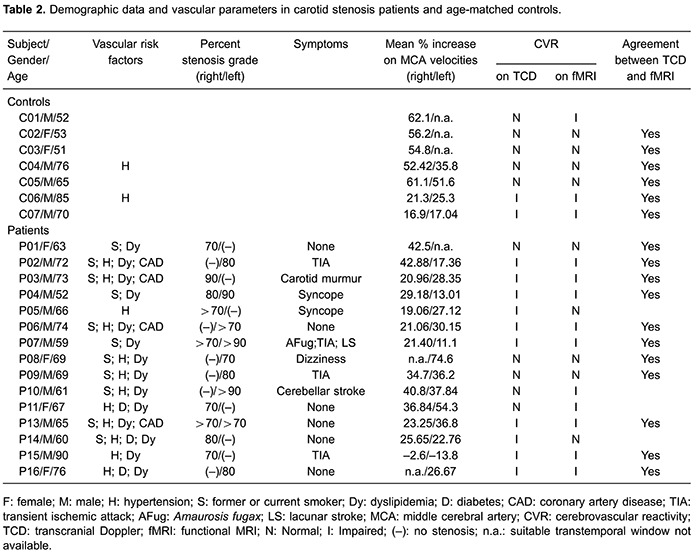

For fMRI, the graphical shape of the temporal variation of delta-BOLD% response during the 3 phases of the paradigm (yellow, red and green) typically consisted of an expressive increase in the signal intensity during the yellow screen followed by a deep undershoot on the apnea time (in the first 10 s of the red screen), and then a second increase while going from the red screen into the first few seconds of the green screen followed by a decrease towards the baseline signal intensity while resuming normal breathing.

**Figure 2:**
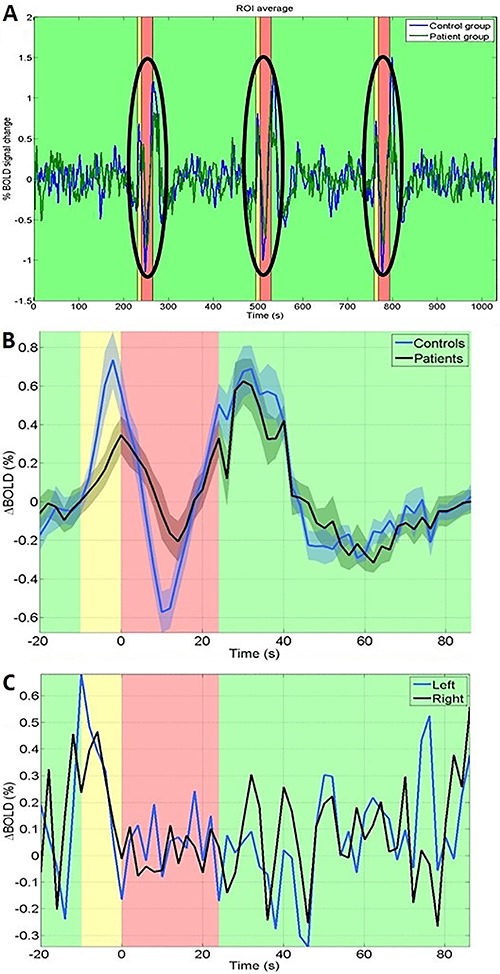
*A*, mean BOLD% signal variation in the time series during paradigm patients and controls. *B*, averaged delta BOLD% signal, which was significantly different between groups (P=0.032, Mann-Whitney test). *C*, visually different BOLD signal curves were observed in both left and right lentiform nucleus of the patient P12, who did not comply with the breath-holding test. In *A*, *B* and *C*, the background colors represent the paradigm tasks during the green (normal breathing), yellow (normal breathing in preparation for holding) and red screen (breath holding).

However, the delta BOLD% signal was significantly different between the groups, particularly in the interval between 4 and 12 s after the onset of breath hold (Mann-Whitney test, P=0.032). The individual analysis of the temporal series of the ROI of all the subjects classified 3 controls and 10 patients as having impaired CVR. Both groups performed similarly for the BHT task, except patient “P12”, excluded for not accomplishing BHT on fMRI (Figure 2).

There was an 85.7% (6/7) observed rate of agreement between TCD and fMRI with a good Cohen's kappa coefficient (κ=0.69) for controls. For patients, the agreement between TCD and fMRI was fair (73.3%; 11/15; κ=0.43). Overall, TCD and fMRI showed a good agreement (77.27%; 17/22; κ=0.54).

## Discussion

The goal of the present study was to assess CVR in patients with CS and age-matched controls through TCD and fMRI using an identical vasodilatory stimulus on both. The delta-BOLD% curve was significantly different between patients and controls. Additionally, there was good agreement between the tests for controls and overall. Within the patient group, the agreement was fair only. Our study is original because: 1) BHT was used for inducting transient hypercapnia in both TCD and fMRI; 2) controls were included; 3) LN was the ROI, and 4) the agreement between TCD and fMRI results was primarily assessed.

We chose BHT because of its low cost, easy performance and overall good correlation to other vasodilatory stimuli (8,26). BHT can cause some concomitant hypoxia during hypercapnia, which may influence the ventilatory response (27) and, additionally, have some issues related to the within-observer long-term reproducibility (28). However, we argue that the short duration of each apnea in our study as well as the fact that TCD measurements were obtained by only one examiner and on a short-term basis have probably reduced those disadvantages.

We included controls to verify whether fMRI with BHT would be able to detect any impairment on CVR, compared to TCD, even in the absence of CS. In our study, the signal averaged time series of BOLD response to BHT in the ROI elicited roughly similar graphical shapes in both groups.

Regarding the graphical shape of the temporal variation of delta-BOLD% during the 3 phases of the paradigm (yellow, red and green), we argue that the increase on the delta-BOLD% in the very last seconds of the yellow phase may have occurred because, in spite of being instructed to avoid hyperventilation, it is possible that subjects may have performed inspiratory movements in a deeper way than the expiratory ones, leading to some degree of CO_2_ retention just before apnea. The curve then decreases within the first 10 seconds of the red screen probably because, at that point, in spite of the progressive increase on the CO_2_ concentration, the concentration of oxygenated hemoglobin is still higher than the concentration of deoxygenated hemoglobin. As the apnea period continues, CO_2_ concentration increases and the concentration of deoxyhemoglobin overcomes the concentration of oxyhemoglobin. So the delta-BOLD% curve goes up again towards the first few seconds of the green screen period, when the concentration of deoxyhemoglobin is still higher as there is a physiological delay for the wash out process. As the normal breathing is resumed, the delta-BOLD% curve goes down and back into a steady stage.

Delta-BOLD% signal was significantly lower in patients than in controls. This may reflect the degree of collateralization, slow flow, as well as an increased baseline oxygen extraction fraction in patients as compared to controls. Additionally, patients showed a slight delay on the time-to-peak, which may suggest some degree of hypoperfusion or even an uncoupling of blood flow and metabolism (29,30). Chang et al. (29) applied BHT in 17 patients with unilateral CS, in which impairment of CVR was evaluated directly by the comparison of the dynamic pattern of BOLD change over the MCA territory between the hemispheres, considering the contralateral hemisphere as an inner control for each patient. In that study, most patients showed a great heterogeneity regarding BOLD curves, which might have resulted either from the wideness of the chosen ROI or from the lack of an outer control. We argue that the role of the contralateral hemisphere as an inner control is not appropriate, as the presence of a microvascular dysfunction in the non-stenotic hemisphere may conceal any eventual difference on the expected BOLD responses leading to a false negative interpretation. This probably could be overcome with age-matched controls inclusion. However, as CVR impairment may result either from macrovascular effects of CS on perfusion as well as from microvascular dysfunctions (31), which may explain the CVR impairment observed in some of our controls (28% by TCD; 43% by fMRI) as this group was matched for age only, but not for vascular risk factors.

LN was chosen as the ROI because of its dense arteriolar irrigation and earlier BOLD response to transient hypercapnia stimuli than other locations (29,32,33). Besides, as a more centered structure, it is less susceptible to peripheral movement artifacts. Another particular interesting point for choosing LN while using BHT as the vasodilatory stimulus is that its neurons are directly involved in the complex network of cortical and subcortical structures integrated by the pons that, in turn, exerts inhibitory effect on medullary respiratory neurons during voluntary apnea (34).

Most of the studies that used TCD and fMRI for CVR assessment in patients with CS or occlusion were primarily aimed to investigate singularities of BOLD curves in response to several paradigms rather than the agreement between the methods (15,16,18,). Even so, two studies with carbon dioxide inhalation demonstrated a good agreement between TCD and fMRI (20,22). In our study, the agreement was overall good, but only fair in patients. This may be due to the small sample or, probably, to the fact that TCD was arbitrarily set as the reference test as PET was not available in our institution. The reliability of TCD for CVR screening in patients with CS is still controversial: few studies have shown a good correlation between CBF velocity in the MCA measured by TCD and the blood flow volume assessed by PET (6,11,12), but the association between increased OEF on PET and impaired CVR on TCD has not been consistently found (35).

In conclusion, our study supports that fMRI with BHT is a feasible and useful tool for mapping CVR in patients with carotid stenosis, as MRI can overcome some of the main limitations of TCD: the operator-dependence, the need for an ultrasonic window and the lack of imaging. Further studies with larger samples of patients and age- and vascular risk-matched controls may provide a more accurate measure of the agreement between these methods.
